# The work of conflict mediation: Actors, vectors, and communicative relationality

**DOI:** 10.1177/0018726721994180

**Published:** 2021-02-25

**Authors:** Boris HJM Brummans, Lise Higham, François Cooren

**Affiliations:** Université de Montréal, Canada, boris.brummans@umontreal.ca, lise.higham@umontreal.ca, f.cooren@umontreal.ca

**Keywords:** agency, communicative relationality, conflict management, conflict mediation work, relational ontology, text, textual agency, third-party dispute resolution, vector, workplace mediation

## Abstract

Mediation is a widely used form of third-party conflict management for which research has primarily focused on the role of mediators. But how are the relations between disputing parties constituted in communication involving written texts, such as official letters or medical reports, during mediation sessions? To gain deeper insight into the communicative dynamics through which third-party disputes are created, sustained, and resolved, this article proposes a new theoretical perspective on mediation that illuminates how human beings and written texts can act as vectors for each other, i.e., how they can make important differences in mediation sessions because they carry or convey what someone or something else is saying, doing, thinking, or feeling and, thus, contribute to composing the nature of disputants’ relations. The value of this vectorial perspective on mediation is subsequently demonstrated through an inductive analysis of video-recorded sessions that took place at an administrative tribunal in Canada. By showing how texts (or their absence) can act as (1) conjunctive vectors that contribute to highlighting disputants’ compatibilities and help them find common ground, or (2) disjunctive vectors that contribute to highlighting their incompatibilities and obstruct their dispute resolution, this article advances the academic and professional literature on the role of communication in conflict mediation work, and reveals significant implications for the study and practice of conflict management in organizations as well as scholarship on relational ontologies.

Over the past 40 years, conflict mediation—defined as a “type of conflict management whereby an outsider or third party intervenes in a conflict, in a voluntary, noncoercive manner, in order to arrest its destructive tendencies” (Bercovitch, 1999: 403)—has been flourishing (Moore, 2014), and organizations have become increasingly interested in Alternative Dispute Resolution (ADR) programs, such as workplace mediation (Bennett, 2013; Bollen and Euwema, 2013; Bollen et al., 2016; Brubaker et al., 2014; Latreille and Saundry, 2014; Putnam, 2007). Whereas arbitration gives the arbitrator the authority to settle a dispute, conflict mediation is typically characterized by an absence of decision-making power on the mediator’s part. Thus, in most western mediation models, conflict settlement ultimately lies in the hands of the disputing parties, as they are supposed to find a solution themselves (for non-western models, see Wall and Dunne, 2012).

Much attention has been given to the work of mediators (see Aakhus, 2003; Glenn, 2010; Heisterkamp, 2006a, 2006b; Jacobs and Aakhus, 2002; Jameson, 2007a, 2007b; Jones and Bodtker, 2001; Kolb, 1985; Kressel et al., 2012; Moore, 2014; Poitras, 2009, 2013; Putnam, 2007; Raines and Choi, 2016), and models have been developed to represent different types of conflict mediation work (see Donohue 1991, 2006). According to many of these models, the mediator’s main task is to facilitate the negotiation between the disputants (Gulliver, 1977). This impartiality puts mediators in a paradoxical position: they are not supposed to have the power to change the conditions of the conflict, yet are also expected to help the disputants find ways to resolve their disagreements (see Aakhus, 2003; see also Silbey and Merry, 1986). As Greco Morasso (2011: 6–7, emphasis in original) puts it:

[A]lthough mediators are crucial in defining the parties’ argumentative discussion, they are far removed from corresponding to the traits of canonical arguers. In fact, mediators are expected to contribute to the discussion without expressing a personal standpoint about any specific resolution, and without advancing any arguments in support of given standpoints. This is their paradox: *they express no standpoint and no argument, yet they are essential for the argumentative discussion in mediation to be fruitfully conducted*.

Because of their commitment to impartiality (see also Cohen et al., 1999; Hale and Nix, 1997; Harrington and Merry, 1988; Heisterkamp, 2006a, 2006b), mediators, like facilitators, work to shape outcomes without providing solutions (see also Cooren et al., 2006). Their interventions need to be perceived as expressing or translating what the disputants are thinking and feeling about the situation they are confronted with, or as steps to move the mediation process forward. Mediation work, in turn, consists of such practices as *asking questions* (Greco Morasso, 2007; Jacobs, 1992, 2002; Van Eemeren et al., 1993); *reformulating* or *summarizing* what the parties are saying, and, in so doing, *redefining* what is said (Greco Morasso, 2007; Jacobs, 2002); *informing* (Jacobs, 2002); and *creating an externalizing conversation* (White, 1989; Winslade et al., 1998), i.e., inviting disputants to produce a story in which the conflicting parties appear as separated from the conflict, or as collateral victims of what is happening.

Thus, the conflict management literature tends to view mediators as the central intermediaries or go-betweens through which disputants can find ways to resolve their disagreements “by themselves.” As a consequence, the literature often does not sufficiently acknowledge that conflict mediation work is a matter of joint relationship (re)building to which the disputants and various other actors (from the Latin *agere*, “to set in motion, drive, drive forward, do, perform”) contribute, as well as how mediators *together with* these actors compose the nature of their relations in the course of communication during mediation sessions.

One specific type of actor that the mediation literature rarely considers is the role of written texts (reports, letters, memos, etc.) in the composition of relations during mediation, even though research in fields ranging from linguistics to organization studies suggests that texts make a difference (Latour, 1996, 1999) in the ways human beings relate to each other, structure interactions, and enact social collectives (see Anderson, 2004; Asmuß and Svennevig, 2009; Brummans, 2007, 2018; Brummans et al., 2020; Castor, 2018; Castor and Cooren, 2006; Chaput et al., 2011; Cooren, 2004, 2008, 2009, 2015a; Fauré et al., 2010; Hall and Butler, 2017; Jahn, 2018; Kameo and Whalen, 2015; Karlsson, 2009; Kuhn, 2008, 2012; Meier and Carroll, 2020; Sergi, 2013; Smith, 2001, 2005; Spee and Jarzabkowski, 2011; Svinhufvud and Vehviläinen, 2013; Vaara et al., 2010; Vásquez et al., 2016). Written texts, for example, dictate rules of conduct, give some people authority while depriving others of it, assist people in managing tensions or making decisions, or give organizations a constitutional basis. Correspondingly, written texts clearly play an important role in mediation work, but few studies have theorized and analyzed how texts co-compose the relations between human actors during mediation.

To address these issues, this article provides a new theoretical lens for investigating the work of conflict mediation, grounded in the idea of *communicative relationality*—how relations are communicatively constituted through a multitude of actors (see Cooren, 2015b, 2018a, 2018b; Cooren and Sandler, 2014; Cooren et al., 2017; Jahn, 2018; Kuhn, 2020; Kuhn et al., 2017). This perspective is useful because it offers conflict management researchers and professionals insight into how and why mediators, disputants, and written texts are able (or unable) to affect the course of mediation. To develop this perspective, this article extends Cooren’s (2004, 2008, 2009) research on *textual agency* by viewing both texts and human actors as *vectors* (from the Latin *vehere*, “to carry, convey”). Viewing texts in this way does not only bring attention to the agency of these actors (to the fact that they make a difference in a given situation), but also to *how* this difference-making is accomplished, namely by carrying or conveying what someone or something else is saying, doing, thinking, or feeling. Hence, the vectorial perspective presented here provides a more nuanced and accurate conception of how the agency of textual actors is brought about in connection with the agency of human actors, and how these interconnections are key to the (re)building of relations between disputants, as part of the wider web of relations between the mediator, disputants, and texts. It reveals how texts as vectors are able to affect the process of mediation as their carrying or conveying abilities interconnect with the carrying or conveying abilities of human beings, who act as vectors as well. This article therefore offers insight into the communicative work through which fields of relations between mediators, disputants, and texts get constituted in mediation.

In what follows, we develop our theoretical lens on the communicative relationality of mediation work. We then show the value of this lens through an inductive analysis of video-recorded mediation sessions that took place at an administrative tribunal in Canada. To conclude, we discuss the implications of this article for the study and practice of mediation work, including workplace mediation in organizations as well as scholarship on relational ontologies.

## A vectorial perspective on the work of conflict mediation

Relational ontologies have been receiving increased attention in fields such as organization studies (Orlikowski, 2007; Vosselman, 2014) and communication studies (Cooren, 2015b; Cooren and Sandler, 2014; Ganesh and Wang, 2015; Jahn, 2018), because they “can facilitate novel ways of attending to social problems” (Kuhn et al., 2017: 4). Here, we build on an ontology that highlights the role of communication in composing relations, referred to as *communicative relationality* (Cooren, 2015b, 2018a, 2018b; Cooren and Sandler, 2014; Cooren et al., 2017; Jahn, 2018; Kuhn, 2020; Kuhn et al., 2017), since it provides a useful starting point for conceptualizing conflict mediation work as a communicative process through which the nature of relations between various actors gets composed in mediation.

As Kuhn et al. (2017) note, those adopting an ontology of communicative relationality presume that relations are made by human and other-than-human actors, but also make these actors in their turn. In the course of their (linguistic and extra-linguistic) enactment, relations “make possible and recognizable the very stuff of the world” (p. 32). Relations can therefore be seen as “trajectories of practice” (p. 32), especially communicative practice, or as the actualization of countless potential courses of action: “Although their enactment tends to develop habitual, territorial defenses that perpetuate some patterns down the line, [relations] could always be accomplished otherwise, and they frequently veer off course” (pp. 32–33). Consequently, Kuhn et al. (2017: 33, emphasis added) suggest, “[o]nly *vectors* of ordinary practice can explain the relations in which we find ourselves”.

While Kuhn et al. (2017) do not elaborate on the concept of vector in their book, we believe it is useful for developing a more nuanced and accurate conception of how actors make differences in situations (their agency) by carrying or conveying what other actors are saying, doing, thinking, or feeling, and this is what defines the effects of their contributions to composing the nature of relations—their *vector effects*, so to speak. If “action entails a multiplicity of hybrid forces at work in concrete practice,” as Kuhn et al. (2017: 36) claim, viewing actors as carriers or conveyers of other actors’ words, actions, and so on not only offers insight into how agency as a “hybrid [(sociomaterial)] and distributed phenomenon” (p. 36) is accomplished in communicative practice, but also offers insight into how this accomplishment enables the co-composition of relational webs or fields that make actors who or what they are.

Following this line of thought, we extend Cooren’s (2004, 2008, 2009) view of textual agency by regarding written texts as key vectors in the constitution of relations. Building on Weber’s (1947) study of texts in bureaucratic organizations as well as Smith’s (2001) institutional ethnographies on how written texts partake in an organization’s or institution’s *objectification*, among others, Cooren’s research decentered traditional analyses of human interactions by highlighting that texts make a difference in such interactions. In keeping with actor-network theory (Callon, 1986; Latour, 1992, 1996, 1999, 2005), the notion of textual agency entails recognizing that texts share their ability to make a difference (agency) with other texts, artifacts, and human beings. For example, Brummans (2007) showed that his father shared his agency with a euthanasia declaration, which participated and significantly affected family decision-making during his father’s final days.

The ways in which written texts operate as agents has been studied across contexts (see Benoit-Barné and Cooren, 2009; Brummans, 2007, 2018; Caronia, 2015; Caronia and Cooren, 2014; Caronia and Mortari, 2015; Cooren, 2004, 2008, 2009, 2015a; Jahn, 2018; Meier and Carroll, 2020; Spee and Jarzabkowski, 2011; Vaara et al., 2010). Yet, how the sharing of agency is accomplished and, in turn, affects the composition of the nature of human relations remains obscure. Understanding the latter is especially important for mediators in certain types of conflict mediation, such as divorce or workplace mediation, which rely more heavily on legal documents, reports, letters, and so on than does community mediation. The conflict mediation literature is relatively silent, though, about the role of written texts, other than the final agreements produced at the end of successful mediations (Herrman et al., 2006; Moore, 2014). Most studies, that is, focus on examining the terms that lead to (written or oral) agreements (e.g., see Olekalns et al., 2010), rather than the texts themselves.

In conflict situations, the nature of the relations between interacting parties is typically characterized by their incompatible interests, goals, ideas, behaviors, emotions, and so forth, as well as their interdependency (see Putnam, 1989; Schneer and Chanin, 1987). Especially in workplace and divorce mediation, albeit also other types, written texts have the ability to affect how the nature of the disputants’ relation gets formed during the course of mediation sessions. Owing to their special attributes, texts are able to convey expert voices or the voice of the law, as well as facts, past events, and so on. In turn, texts may hold people accountable for past actions or affect the definition of people’s identities during mediation.

It is therefore useful to investigate types of mediation that rely on written texts through a vectorial lens, because it can reveal how the relation between the disputing parties is enacted through reading, invoking, and discussing reports, letters, and so on that carry or convey the words, actions, thoughts, and feelings of others in the communication between the disputants and the mediator, who *themselves* also act as vectors by carrying or conveying the words, actions, thoughts, and feelings of others. More precisely, a vectorial perspective can reveal how texts contribute to *transforming* disputants’ incompatibilities into compatibilities during mediation sessions, or vice versa. For example, when reading their written statements about what disputants appreciate about each other during divorce mediation (texts created on the mediator’s request), these texts may act as *conjunctive* vectors that reconnect the disputants by helping them become aware of the compatibilities that led to their marriage, as can be seen in the film *Marriage Story* (Baumbach, 2019). However, texts may also act as *disjunctive* vectors by accentuating differences between the disputing parties. Similarly, a written text may act as a conjunctive vector by attesting the existence of a contractual agreement between an employee and an employer during workplace mediation in the case of employee termination. By giving the parties a chance to revisit their official agreement, the document enables them to become aware of their rights and obligations and, thus, helps them negotiate a severance package.

As vectors, then, texts actively participate in the work of various types of conflict mediation. Their vector effects or abilities to transform the nature of the disputants’ relation depend, though, on how they are mobilized, appropriated, interpreted, and so on by the mediator and the disputants, who also become vectors themselves. In *Marriage Story*, for example, one of the disputants refuses to read the statement she has written because she does not like what she wrote and does not want to hear her partner’s statement. A text may therefore have a disjunctive vector effect in terms of transforming disputants’ compatibilities into incompatibilities through its *absence*. In such cases, the fact that a key textual carrier or conveyor is unable to act affects the constitution of their relation; as the human actors’ ability to rebuild their relation depends for an important part on the vector effect of the absent textual actor in this situation, they cannot move forward and overcome their differences. In other cases, however, an absent text may have a conjunctive effect, depending on the human actors’ mobilization, appropriation, and so on.

To show how our vectorial perspective on mediation work can produce valuable insights for conflict management researchers and professionals, as well as, more broadly, scholars interested in relational ontologies, we will now present an empirical study of this interplay of human and textual actors’ vector effects.

## Methods

### Type of conflict mediation work studied

In this study, we analyzed mediation sessions that took place at an administrative tribunal in Canada, because this institutionalized type of mediation relies strongly on the mobilization of written documents. As Kuttner (2017) notes:

Administrative tribunals make decisions on behalf of federal and provincial governments when it is impractical or inappropriate for the government to do so itself. Tribunals are set up by federal or provincial legislation; this is known as “empowering legislation” . . . They make decisions about a wide variety of issues, including disputes between people or between people and the government . . . Their decisions may be reviewed by the courts. Because they engage in fact-finding and have the power to impact personal rights, tribunals are often seen as “quasi-judicial.”

As institutions, tribunals depend on the interpretation and application of laws written into texts. Rather than merely observing the “weighty presence” of texts in this context, this setting is particularly suitable, we will show, for analyzing how texts contribute to composing the nature of relations between disputants by carrying or conveying the words, actions, thoughts, and feelings of particular actors.

The tribunal that formed the setting for our empirical research referred to its mediations as “conciliations” and treated conciliations as mediations, as is often the case. Likewise, *The Jackson ADR Handbook* (Blake et al., 2016: 270) states that conciliation “is a facilitative dispute resolution process in which a neutral third party seeks to assist the parties to a dispute to reach a settlement.” As it is “virtually indistinguishable from mediation” (p. 270), we use the terms synonymously in this article.

Conciliation was created to offer citizens an alternative way to contest a decision that concerns them, made by a public institution that focuses on social aid, transportation, and so forth. In Donohue’s (1991, 2006) classification of types of conflict mediation work, this type of mediation follows the *disputant-control model*, as the disputants have absolute control over the outcomes of the mediation. The mediator is supposed to remain impartial and has no power to impose a solution on the disputing parties (the citizen(s) and the public institution in question). This means that citizens and/or the public institution can always decide to go to court (i.e., to have a court hearing), if they do not manage to find a way to resolve their dispute.

Our methodological decision to focus on this type of mediation work limits the transferability (Lincoln and Guba, 1985) of our findings to other types of mediation and other contexts. As we already noted, though, texts play a key role in workplace or divorce mediation, too. Hence, the aim of our study of conciliations is not to provide insight into the typical ways in which texts contribute to constituting disputants’ relations across a range of types of mediation; rather, we aim to show what theoretical and practical insights conflict mediation researchers and professionals can gain from using our vectorial perspective in settings where texts clearly affect disputants’ relations through their conjunctive or disjunctive vector effects.

### Data collection and analysis

The data for this inductive study were collected by video-recording 27 mediation sessions that took place between November 2017 and February 2018. In these sessions, citizens contested past decisions made by public institutions (or the lack of action taken since past decisions were made). And in many of these sessions, both the citizens and institutional representatives invoked different types of written texts (medical reports, medical prescriptions, legal documents, official letters from institutions, handwritten notes, etc.). Hence, texts were not simply ubiquitous, but affected disputants’ relations by carrying or conveying other actors’ words, actions, thoughts, and feelings in these sessions.

Each person who participated in our research gave their written consent prior to the video-recording. In total, 7 mediators (4 women and 3 men) participated. Of the 27 citizens who participated, 13 were female and 14 were male. Out of the 27 institutional representatives, 26 were female and one male. To protect the citizens’ identities, we decided to use first-name pseudonyms in our analyses. To protect the public institutions’ identities, we do not mention their official names or the names of their representatives either, but rather refer to “the institution” as well as “the institutional representative.” We also do not mention the mediator by her or his name, and simply refer to “the mediator.”

Each session lasted between 25 and 80 minutes. We recorded the sessions by using two GoPro cameras that were installed at different positions on the table. The cameras were small, but visible, and gave us a panoramic view of the room. We were thus able to record the disputing parties (citizens were sometimes accompanied by a family member and/or a lawyer) and the mediator (conciliator) while they were talking to each other, and to record how the interlocutors mobilized different kinds of written texts. After recording the sessions, the two recordings were edited side by side, which enabled us to observe the actions of all the participants, including the texts. Subsequently, each session was transcribed by paying attention to brief intervals/pauses, overlapping speech, and so on. We included our descriptions of how texts were mobilized as well as mediators’ and disputants’ nonverbal behavior in double parentheses.

Of the 27 mediations, 5 were conducted in English and 22 in French. We translated the transcripts of the French sessions into English. We realized that the latter would make it impossible to track interlocutors’ precise speech overlap. However, the aim of our analysis was not to “describe the stable practices and underlying normative organizations of interaction by moving back and forth between the close study of singular instances and the analysis of patterns exhibited across collections of cases” (Sidnell, 2016: 1), which is the aim of conversation analysis; let alone to conduct a full-fledged multimodal conversation analysis, which aims to describe “the diversity of resources that participants mobilize to produce and understand social interaction as publicly intelligible action, including language, gesture, gaze, body postures, movements, and embodied manipulations of objects” (Mondada, 2019: 47). Although conducting the latter in future studies would be useful, the goal of this exploratory research was to gain a first insight into how textual and human actors make differences in situations by carrying or conveying what other actors are saying, doing, thinking, and feeling, and through their vector effects, contribute to the composition of the nature of relations. Hence, the method we used to analyze our data parallels the method other scholars have used in studies of communicative relationality (see Cooren, 2018a; Cooren et al., 2017; Kuhn et al., 2017; Martine et al., 2019).

Specifically, we first collectively read our transcripts while watching the video recordings to uncover potential patterns in how disputants and mediators across the recorded mediation sessions read, invoked, debated, and so on written texts in their communication. In so doing, we observed that texts participated in the sessions to varying degrees. We therefore decided to set aside 13 sessions in which texts played a secondary role, such as sessions in which a citizen arrived with a pile of documents in such disarray that it stalled the session, or sessions in which citizens did not understand the mediation procedure at all. This left us with 14 sessions that either ended in a settlement (3), a follow-up session (7), or a court hearing (4). For each of these sessions, we examined how the mobilization, appropriation, and interpretation of specific present or absent texts by the human actors affected the constitution of disputants’ relations. That is, in these sessions, we analyzed how the mobilization, appropriation, and interpretation of these texts’ ways of carrying or conveying words, actions, and so on had a conjunctive effect in terms of highlighting disputants’ compatibilities and helping them find common ground, or a disjunctive effect in terms of highlighting their incompatibilities and obstructing their dispute resolution (see Table 1).

**Table 1.** Vectorial analysis of 14 cases.

Mediation outcome	Case	Textual actors’ disjunctive vector effects	Textual actors’ conjunctive vector effects	Case description
Settlement	Anna		Signed document stating that Anna is living martially with her partner	The institutional representative focuses on the letters that Anna has signed, which state that she and her ex-partner (now deceased) were living as a couple. The disputing parties ultimately decide to settle, meaning that Anna agrees to reimburse sums that had been allocated to her ex-partner, and spent by him, of which she was not aware.
	Luc		Absent documents justifying the need for a social allowance	The institution demands reimbursement of social aid payment, because Luc does not have the documents justifying the need for this allowance. Luc will have to provide documents for this allowance if he goes to court. He chooses to settle in mediation, and they agree on the amount to be reimbursed.
	Serge		Decision letter stating that Serge needs to pay back a social allowance he was not supposed to receive	Serge does not contest the institution’s decision letter (he must pay back the allowance he was not supposed to receive); only the amount of the monthly reimbursement. He asks to lower the amount. The agreement is settled by lowering the monthly amount he needs to pay back and spreading it out over a longer period of time.
Follow-up mediation session	Charles		Several documents (paychecks, etc.)	Charles had two simultaneous employments, which has caused misunderstandings. During their interactions with the institutional representative, Charles and his lawyer try to clear up these misunderstandings. They agree to sort out the documents (paychecks, etc.) and send them to the representative, so she can have a clearer understanding and possibly agree to settle.
	Curt (Case 2 in our analysis)		Medical reports + one to-be-produced letter	Of the 29 medical reports Curt has, one is preventing him from receiving the compensation he is entitled to. Curt, Curt’s lawyer, the mediator, and the institutional representative agree to ask Curt’s doctor to write a letter explaining her mistake, so a settlement can be reached.
	Gaston	Initial accident report	To-be-produced employer’s letter	The institution contests that Gaston’s pain was caused by his accident. The pain only appeared two days after he had the accident and was therefore not mentioned in the medical report on the day of the accident. The institutional representative suggests to get the report revised, while Gaston offers to have his employer produce a letter testifying that he was not able to work due to the pain the day after the accident.
	Henry		To-be-produced medical expertise report	Henry needs to get a new medical expertise report to confirm the connection between his injury and the accident in which he was involved. The institution will make a decision based on this report.
	Louis	Medical expertise report	Absent medical counter-expertise report	Louis was injured while helping a person who had had a car accident. He claims that his medical expertise report was not written by the person who signed it and, thus, that it is false. He has had a counter expertise report prepared. The institutional representative has not received it, so no decision can be made yet.
	Patty		Absent documents + to-be-produced medical exam report	The institutional representative is missing some documents that Patty claims to have received and thought to have sent. Moreover, Patty needs to do another medical exam and the report needs to be sent to the institution, before a settlement can be reached.
	Paul	To-be-produced medical expertise report	To-be-produced medical expertise report	The disputing parties agree that Paul needs to get a new medical expertise report and send it to the institution, so his case can be discussed.
Court hearing	Alba	Several documents stating Alba’s partner’s status		The disputing parties do not agree on Alba’s partner’s status (husband, legal partner, etc.) as it is stated in several documents. This status would make a difference in terms of the amount of her monthly allowance. Because they cannot come to an agreement about this status, they decide to go to court.
	Bud	Absent written proof that Bud was refused student aid		Bud is not able to produce written proof that he was refused student aid. The absence of this document brings the session to a halt. Bud chooses to go to court.
	Doug (Case 1 in our analysis)	Medical expertise report + absent doctor’s letter		Doug wants a letter from the doctor, designated by the institution, stating that he can stop his vehicle whenever and wherever he wants (due to the pain in his leg caused by the accidents he had). He also claims that the medical expertise report is biased. He claims that he wants to go to court. The mediator gives him 30 days to think about this.
	Tom	Absent medical expertise report		The institutional representative needs a medical counter-expertise report to prove the causal connection between Tom’s accident and the injuries he incurred. Tom does not want to have one made, so they decide to go to court.

We selected two cases out of the 14 sessions that offer the richest, most compelling examples of this interplay of vector effects to provide detailed insight into these communicatively relational dynamics (see next section). To create as much contrast as possible between the cases, we purposively selected one mediation session that (presumably) ended in a court hearing (Case 1) and one that ended in a follow-up session (Case 2). We also selected these sessions because they show how the presence or absence of a key text can have a disjunctive (Case 1) or conjunctive (Case 2) effect on the nature of disputants’ relations. A final reason for selecting these cases was that a different mediator acted in each of them.

Evidently, by zooming in on the interplay of human and textual actors’ vector effects, we did not pay close attention to other factors, such as gender, race or ethnicity, social class, education, and so on, although these factors clearly affected the constitution of disputants’ relations as well. It would be important for future studies to take such factors into account when analyzing mediation sessions from a vectoral perspective.

## Analysis of vector effects in conflict mediation work

### Case 1: How disputing parties become entrenched through vector effects

Doug was involved in a car accident. He was gradually returning to work as a car courier when he became the victim of another accident. In the mediation we analyze here, Doug requests that a letter be written by a doctor, stating that his current physical condition allows him to safely drive a vehicle and stop it whenever needed. If this letter cannot be produced, he would like to receive financial compensation for his second accident and financial support for professional retraining. He also claims that the medical expertise report has omitted to mention certain aspects of his condition—it indicates that he can return to work, while he claims the contrary.

The meeting is very tense. Although Doug clearly states that he wants to go to court at the end of the session, the mediator offers the possibility of doing a follow-up session and invites him to produce new evidence. In the next excerpt, Doug is in the middle of reading a two-page text he wrote in anticipation of this session. Before starting to read it, he calls this text “my perspective on the whole thing”:

73 Doug Today, first of all, I would like to make one request, and if the other party can

74  accommodate it, it won’t be necessary to have this reconciliation to proceed

75   or go to a tribunal here, subsequently. (.) I’m requesting that the [name of

76   institution] do one thing that closes this case and allows me to take any

77   courier job available with the stated capability based on the assessment of

78   their position. So, if I’m ever involved in an accident anywhere on the public

79   road, as a result of my depreciated capabilities that trigger a sciatic attack in

80   my right leg, I can give a copy of this letter to the investigating officer at the

81  scene of the accident or produce it for future reference in court. If this is

82  possible and can be arranged, I’m willing to take the risk and return to work.

83  (.) The purpose is because there is a discrepancy with regard to my opinion of

84  what I conclude is possible to happen during the operation of my vehicle at

85  work and the doctor’s contrasted opinion based on his findings assessed by

86  examination and all the scientific data made available to him. (.) If he can

87  prove that there is a preponderance of irrefutable scientific evidence that

88  prevents me from getting involved in an accident, he should be willing to

89  back it up with his signature.

Doug is asking for a letter that would protect him if he were to be involved in another accident. If he gets into another accident because of what he calls his “depreciated capabilities that trigger a sciatic attack in [his] right leg” (lines 79–80), he would be able to “give a copy of this letter to the investigating officer at the scene of the accident or produce it for future reference in court” (lines 80–81). In this case, the relation between him and the law would change through the presentation of this letter, Doug appears to presume, for it would exonerate him from any responsibility. The letter would have a significant vector effect, because it would enable Doug to make its author—the doctor designated by the institution in question—say that “there is a preponderance of irrefutable scientific evidence that prevents me from getting involved an accident” (lines 87–88). Thus, the letter would not only serve as Doug’s protector in the event of an accident, but also as proof that the doctor was wrong. It would make the doctor say that it was safe for Doug to drive a vehicle, while the accident would provide evidence to the contrary. Through its vector effect, the letter could therefore alter the doctor’s reputation, making him appear incompetent—a point on which Doug keeps insisting throughout the session. Moreover, this letter could also change Doug’s legal identity by making him unresponsible for any accident that happens while driving his vehicle.

After Doug has finished reading his statement, the mediator (Med1) attempts to summarize Doug’s expectations by writing them on big white sheets of paper hanging on the wall. One of these expectations concerns the letter Doug is asking for, as well as several other requests he is making. After she has completed this task, she says that “the facts are on the table” (line 330) and then asks R1, the lawyer who is representing the institution, to present their position. In the next excerpt, R1 comments on what the mediator has written on the white sheets:

351 R1 As for the letter that you are asking ((R1 looks at the sheets, while Doug

352  looks at her)) uhm, I cannot today, and I want to clarify that first, I cannot

353  ((putting hand on chest)) commit myself to ask for a doctor to sign ((R1 does

354  not look at Doug, but at the writing on the sheets, and points to them)) such a

355  letter ((turns to Doug)). Uhm, and we already have the [written] expertise

356  from Dr. D. stating that you are able to go back to your work, and this is the-

357  the medical evidence that we have right now so-

The letter Doug is asking for cannot be provided, according to R1, as the latter cannot commit herself to ask a doctor to write it. To justify this refusal, she mentions what she calls “the [written] expertise” (line 355) provided by Dr. D. (the doctor about which Doug keeps complaining). According to Dr. D.’s report, R1 notes, Doug is “able to go back to [his] work” (line 356). While Doug is expecting a new letter from this doctor, R1 highlights the existence of past expertise, of which Doug is aware—a written expertise that defines Doug as “able-bodied.” R1 accentuates “the medical evidence” (line 357) by focusing on a document that presents Doug as being capable of driving his vehicle, without adding the qualification that “there is a preponderance of irrefutable scientific evidence that prevents [him] from getting involved an accident,” as Doug requested. In other words, the written expertise, highlighted by R1, underlines the stark incompatibility between Doug and the institution-via-R1.

When Med1 asks R1 to read this evidence, R1 looks through her files and finds Dr. D.’s report, which she starts to read out loud:

400 R1 Uhm, yeah, at the end of the page, the doctor states that “considering the

401  subject’s complaints, and considering our objective examination that showed

402  no active lesion, we can note that, in all likelihood, unless other information

403  is provided to us, that the new event of December 8, 2016 did not aggravate

404  the patient’s clinical condition resulting from the March, no that’s 17 to, the

405  accident of March 17, 2016.” Uhm and after that he says “that there is not any

406  functional limitation nor does it require treatment.” So, he maintains- he

407  maintains the position that he had for the- ((turns to Doug))

408 Doug So, basically what he’s saying is that the accident of eh, the last accident

409  ((R1 nods))

410 Doug that was an attempt by me to defraud the [name of institution] of benefits

411  because what he’s saying is that “you did not have an injury, or whatever

412  happened, there was no reason for you to be off work, or free to be treated”

413  that’s what the doctor is saying

414 R1 ((turning from Doug to the white sheets)) What=

415 Doug =So what the doctor is in essence, is accusing me of committing a criminal act

416  and that’s [punishable by law

417 R1  [I-

418 R1 I don’t think ((chuckles)) that’s what he’s saying at all. I think uhm, what the

419  doctor is trying to- to do is to evaluate ((turning to Doug)) if you have any

420  limitation or restriction resulting from both of your accidents and uhm he

421  compares your uhm condition to the requirements of your actual work that we

422  have, like the description of the task ((turning pages of her document)) uhm

423  that is on page 175. So he compares the requirements of your ((turning to

424  Doug, then back to her reading)) job and your condition, and evaluates if you

425  are on the day of the examination fit to return to work ((looking at Med1)). I

426  think that’s what the doctor is uh saying in this conclusion, uhm and as for the

427  letter that you were requesting ((looking at the sheets while Doug is looking

428  at Med1)) uhm, this expertise states that you are fit to go back to your work

Seen through a vectorial lens, Dr. D’s position, as read by R1, becomes something different in Doug’s mouth, showing the competing vector effects of R1’s and Doug’s verbal actions: “So, basically what he’s saying is that the accident of eh, the last accident that was an attempt by me to defraud the institution of benefits because what he’s saying is that ‘you did not have an injury, or whatever happened, there was no reason for you to be off work, or free to be treated’ that’s what the doctor is saying” (lines 408–413). Dr. D.’s voice is carried or conveyed by two different vectors: through R1’s reading of the report, Dr. D.’s voice is presented as a professional expert voice, whereas through Doug’s reading, Dr. D.’s voice is an accuser’s voice (“So what the doctor is in essence, is accusing me of committing a criminal act and that’s punishable by law,” lines 415–416).

R1 immediately contests how Doug conveys Dr. D.’s voice as it is textualized in the medical report, and tries to reaffirm what she believes the doctor is saying. By mobilizing the report as a disjunctive vector, she again emphasizes the strong incompatibility between Doug and the institution she is representing. R1 emphasizes that the doctor is actually comparing what she calls “the requirements of [Doug’s] job” (lines 423–424) as indicated on page 175, and “[Doug’s] condition” (line 424). Beyond claiming that the doctor is not accusing Doug of anything, she accentuates the report’s factuality. Therefore, it is not only the doctor who shows that “[Doug is] fit to go back to [his] work” (line 428), but also, more specifically, the comparison between the job expectations and Doug’s physical condition, which the report’s vector effect enables.

In this battle of interpretations (which is a battle of vectors through which different textual and human actors end up talking and acting), we see how the nature of the relation between the protagonists changes, depending on what the doctor’s report becomes in their mouths. In Doug’s mouth, the report transforms him into a fraudster or even a criminal, while the doctor and the institution both become accusers who treat him unfairly. In R1’s mouth, however, the same report repositions Doug as an ordinary citizen whose condition was fairly assessed, while the doctor, acting on behalf of the institution, is merely an evaluator doing his job.

As the session proceeds, both parties appear to agree that no solution can be found. Yet, Doug tries to contest Dr. D.’s medical report one last time:

847 Doug On page 291, here where she says, this is my problem, OK? And I’m asking if

848  you saw this information ((looking at R1)) and you are saying- you’re saying

849  you weren’t cognizant of- of it, so- so she asked me ((pointing to Med1)) if I

850  could provide it, in the file, so I’m saying, did you read this part of the file?

851 R1 I can read it now if you wish me, for me

852 Doug XX

853 Med1 [It’s page 291 on the first paragraph

854 Doug [This, yeah, that’s the most important, this is a pivotal piece of information of

855  the file ((tapping on the document with his fingers)) that will take me to the

856  tribunal, as it relates to public safety on the road, and operating a motor

857  vehicle in terms of the residual uhh effect of my accident.

858 R1 Well it- it says that “that there is a postural central XXtion at the ((looking

859  toward Doug)) L4 and L5 [lumbar vertebrae]”

860 Doug Yeah it doesn’t conclude there, it goes further, it says that it affects me more

861  on my right side than on my left

862 R1 Yes, I- I- I understand that ((looking at him))

863 Doug But you haven’t mentioned that, that’s a pivotal thing of this whole thing

864  because my arms are pretty strong ((flexing his muscles)) and I’m a pretty

865  healthy person

866 R1 Listen the- the- the doctor had this information here, he- he- he states it here

867  in the expertise. Now if he had this information and he comes to a certain

868  conclusion, that’s the conclusion that he- he came to, uhm I don’t think he

869  omitted anything in his conclusion

870 Doug OK, that’s your opinion

871 R1 OK

872 Med1 So would you

873 Doug So let’s prove it hard

874 Med1 Yes we have on the table an option, which is yours to take or not ((turns to

875  the white sheets on the wall)), which states that taking into consideration what

876  you have mentioned today, there is an option that they pay you three days of

877  treatment, two months of reimbursement of indemnity, and they give you

878  severity one [a degree of injury established according to a list of injuries used

879  to determine the amounts of reimbursement and indemnity], based on what

880  you’ve mentioned, and with the interest, that’s an option. So if you wish to

881  conclude with this option, then we can draft it and if not, uhm I’ve put a

882  mention that you would like to have the [court] hearing, so you have 30 days

883  to decide.

In a final attempt to convey his side of the story, Doug asks R1 to read what he presents as a key passage of the medical report—a passage that, he says later, “will take [him] to the tribunal” (lines 855–856). R1 responds that she can read it, which is what she does on lines 858 to 859: “there is a postural central XXtion at the ((looking toward Doug)) L4 and L5 [lumbar vertebrae].” Doug then claims that what is important here is the conclusion the report draws from this observation, which is that this “affects [him] more on [his] right side than on [his] left” (lines 860–861). Although R1 acknowledges that she understands this, Doug accuses her of not having mentioned this “pivotal thing” (line 863). By selecting this passage, Doug tries to make it say (and make R1 say) that his condition is actually recognized by the doctor and that “it relates to public safety on the road, and operating a motor vehicle in terms of the residual uhh effect of my accident” (lines 856–857). Dr. D. came to the conclusion that it was safe for Doug to go back to work as a car courier, but Doug tries to make the report (and its author) say that he is actually unfit to return to work.

Here, our vectorial perspective reveals how Doug mobilizes this specific passage as an ally that could help him win his case. Indeed, this passage (and therefore also the doctor who wrote it) does acknowledge that Doug is injured (that he is affected on his right side), contrary to what the doctor claims in the conclusion of his report. Doug seems to acknowledge the potential vector effect of this part of the report when he points out that it is what “will take [him] to the tribunal” (lines 855–856). For him, this passage is important because it has the potential to affect the nature of his relations with the institution-via-R1, with Dr. D., with Med1, and even possibly with court officials, if he decides to go to court. In R1’s mouth, though, this passage is simply reinscribed as a condition of which the doctor was aware. In his report (“Listen the- the- the doctor had this information here, he- he- he states it here in the expertise,” lines 866–867), this condition did not prevent him from drawing the conclusion he drew (see lines 867–869). Thus, in R1’s mouth, the doctor claims Doug is fit to return to work through his report, again accentuating the seemingly insurmountable incompatibility that lies at the heart of the relation between Doug and the institution R1 represents.

### Case 2: How disputing parties start to collaborate through vector effects

In the second conflict mediation session we analyze in detail, Curt, who is accompanied by his lawyer (“Curt law”), has contested a public institution’s decision regarding his medical situation. Curt was involved in a car accident while living abroad. He has been disabled for more than a decade and moved back to Canada a few years ago. Despite filing for a disability pension again and again by systematically providing his updated medical report to the institution (he claims 29 times), Curt has only just now been granted the “permanently disabled” status. Hence, he is claiming the appropriate retroactive compensation for which he is eligible, according to his physician, from the date of the first report the physician filed. All but one of the physician’s 29 reports indicate that Curt was disabled for 6 to 12 months. This report is an outlier that prevents the institution (represented by R2) from granting Curt the “permanently disabled” compensation from the date of the first report.

At the point where we enter the mediation, these issues have been discussed. Now, all the disputing parties as well as Med2, the mediator, are reflecting on how to correct this situation through the production of a new letter:

450 R2 But you just kept the same doctor, there is a continuity, so I think it would

451  be interesting if you had a document from this doctor saying she has=

452 Curt law =That the evolution of the condition whatever, the same perception

453 R2 Yeah like she thought it would get better but it never=

454 Curt =But can-?

455 R2 Like it never changed really, like your condition stayed

456 Curt Can one of you guys write the letter?

457 Curt law Yes yes I will

458 Curt OK, yeah, thank you, ‘cause I don’t wanna say the wrong [things

459 Curt law  [Not for her, but

460  letter to

461 Med2 To

462 Curt law Her

463 Curt To explain

464 Med2 To explain the why and

465 Curt law ((smiles))

466 Med2 What we need to- what the [institution] needs

This is the first occurrence of an emerging collaboration between Curt, his lawyer, R2, and Med2 through the to-be-produced text (R2: “But you just kept the same doctor, there is a continuity, so I think it would be interesting if you had a document from this doctor saying she has,” lines 450–451). This shows the to-be-written letter’s potential vector effect—its potential ability to transform the conflictual nature of the disputants’ relation, depending on how the text is going to be carried or conveyed (mobilized, appropriated, and interpreted) by the various human actors involved. According to R2, the fact that Curt has kept the same physician—a fact attested by the vector effects of many documents in his file—indicates that he has not been “shopping around” to find a doctor who would give him the document he needs to obtain his “permanently disabled” status. These documents therefore act as multiple vectors that carry the evidence that Curt is not “playing the system,” so to speak. From a vectorial perspective, these documents are key to the establishment of a relation of trust between the parties, for they carry the proof that Curt is acting in good faith.

As we see, R2 subsequently tries to find a way to reduce the weight/importance of the medical report that states that Curt is not disabled. She wonders if a new document from Curt’s physician (in the form of an official letter) could make *the* difference in terms of changing how Curt and R2 (and the institution she is representing) are currently related through the anomalous medical report (R2: “it would be interesting,” lines 450–451). Technically, R2 is indeed bound by this medical report that prevents the institution R2 represents from granting Curt his “permanently disabled” status. Because of this report’s disjunctive vector effect, both R2 and Curt are stuck in a position of adversity, for it positions R2 as an obstacle to Curt’s attempt to get this status. What can be seen here is how R2 still seems to be toying with the idea of a new letter, speculating on this new document’s potentially conjunctive vector effect—its ability to counter the disjunctive vector effect of the outlier report.

Curt asks if someone else would be willing to write a letter to his physician, explaining “the why and what” (lines 464–466) that needs to be put in this official letter. He seems afraid that he will “say the wrong things” (line 458) and appears to anticipate the potential vector effect of the official letter in transforming his relation with the institution-through-R2. Curt’s lawyer agrees to write this “why and what” letter to Curt’s physician and ask her for the official letter that, everyone agrees, is necessary to resolve this dispute. The physician’s official letter is to reinterpret the medical report that precedes it, to make it say something else, thus bringing an end to the conflict between Curt and the institution on behalf of which R2 speaks and acts.

What is interesting here is how well Curt, his lawyer, R2, and Med2 collaborate to imagine and create the content of a new text that is supposed to correct the problems caused by another text. The official letter’s vector effect emerges through each human actor’s intervention: R2 first mentions the letter (“a document from this doctor,” line 451). After this, Curt’s lawyer and R2 start completing each other’s sentences (lines 452–453) until Curt himself jumps in (line 454), and then Med2 starts to participate (line 461). Notice how Curt’s lawyer smiles (line 465) as the mediator takes over and explains what the “why and what” letter to Curt’s physician should look like. She is pleased, it seems, that the mediator agrees with this course of action. In fact, Curt, his lawyer, R2, and Med2 all appear to be pleased to have found a way to resolve the dispute. The official letter they want Curt’s physician to write, they seem to realize, can become their “savior” or “liberator” in that it transforms the existing incompatibility between Curt and the institution-via-R2 into a compatibility.

From a vectorial perspective, it is noteworthy how the physician’s official letter can act as a vector through Curt’s, his lawyer’s, R2’s, and Med2’s respective contributions to the mediation, as well as how the nature of the disputants’ relation begins to change through this letter (still fictive at that point). While it has yet to be written by Curt’s doctor (and the hope is that she will agree to do it, because this will make the letter exist “on paper,” thereby increasing its potential conjunctive vector effect), its form and content is progressively anticipated and defined. The new letter’s existence is in-the-making, a matter of degree, in this key episode of the mediation. Each human actor’s respective contribution becomes the means by which this textual actor can change the nature of Curt–R2’s relation. Curt, his lawyer, R2, and Med2 themselves are therefore also key vectors through which this letter begins to exist.

Although the letter has not yet materialized beyond their discussion, its emerging existence in their turns of talk appears to be the beginning of the dispute’s resolution. The new letter is presented as the vector through which Curt’s doctor will be able to requalify the problematic report—to make it say something different. In her letter, the physician could indeed write that “she thought it [Curt’s condition] would get better” (line 453), but that “it never changed really, like your condition stayed” (line 455). The outlier report would consequently be reduced to the mere expression of the physician’s hope, while before it revealed an essential fact. Hence, the new letter would ensure that the anomalous report conveys the physician’s “wishful thinking,” rather than defines Curt’s condition. Whereas the problematic report acted as a disjunctive vector, an obstacle to the resolution of this dispute, the new letter becomes the conjunctive vector through which things can move forward. The letter is meant to flatten the previous medical report, to change how Curt and the institution are related, and to grant him his “permanently disabled” status retrospectively, since his return to Canada.

The next excerpt, taken from the same session, further illustrates these kinds of communicatively relational dynamics. In it, the human actors describe the letter’s future effect in the dispute’s resolution.

734 Curt law Good, so (.) we’re gonna (.) ask the doctor

735  (0.5)

736 Curt To write the letter

737 Curt law Yep

738 Curt And then when do we go for, what (.) eh what do we do from- from when I

739  get the letter from the doctor right?

740 Curt law I’m gonna send it to you, Ms. R2

741 Med2 Yeah

742 Curt law and from there

743 Med2 She will submit it to (.) she sees, you’ll see what [XX your [institution]

744 R2    [Yeah. We have a team

745  we discuss that

746 Curt OK

747 R2 and I discuss your file with my team and I can see from there, and I don’t

748  know if you want to keep the file on [XX

749 Med2   [Yeah we’ll keep it open

Here, the collaboration between Curt, his lawyer, R2, and Med2 continues as they discuss how to organize the letter’s production: Curt’s lawyer initiates this organization by stating in line 734: “Good, so (.) we’re gonna (.) ask the doctor,” and Curt finishes her sentence by saying: “To write the letter” (line 736). The physician’s letter is expected to act as vector in the work of conflict mediation in two ways: working together on the production of this emerging letter unites Curt, his lawyer, R2, and Med2 around a common object; however, the future letter’s vector effect goes beyond mere unification, because it is expected to act as the missing piece in the puzzle to resolve this dispute by carrying the official evidence the institution requires to grant Curt his “permanently disabled” status and the appropriate compensation. The letter is to erase the incompatibility between Curt and the institution altogether. Tailored by the participants in the mediation session and (hopefully) by Curt’s physician later on, the new letter becomes, in other words, an *obligatory passage point* (Callon, 1986) between Curt and the institution; it will officialize the agreement between the disputing parties in a way that satisfies the institution’s administrative requirements. Noteworthy, in this regard, is that the disputing parties’ mere oral agreement would not suffice; the conjunctive vector effect of an official written text is required to accomplish this conflict’s resolution.

## Discussion

Mediation is a widely used form of third-party dispute resolution. Thus far, however, conflict mediation work has been neither conceptualized nor empirically analyzed as a matter of joint relationship (re)building to which different types of actors contribute. To address this issue, we developed a vectorial perspective on mediation, grounded in the notion of communicative relationality (see Cooren, 2015b, 2018a, 2018b; Cooren and Sandler, 2014; Cooren et al., 2017; Jahn, 2018; Kuhn, 2020; Kuhn et al., 2017). This new perspective enabled us to conceptualize the role of textual actors in relation with human actors in mediation work by explicating how texts can become conjunctive or disjunctive vectors during dispute resolution. We then showed the value of this perspective based on an inductive analysis of video-recorded mediation sessions at an administrative tribunal in Canada. As we will discuss next, the theoretical and empirical insights presented in this article are useful for conflict management researchers and professionals, including those interested in studying or practicing mediation in organizations, as well as, more broadly, scholars interested in relational ontologies.

First, this article has important implications for the conceptualization of conflict mediation work. As mentioned, the conflict management literature positions mediators as the central actors through which disputes get resolved, but also paradoxically as mere intermediaries or go-betweens through which disputants can find ways to resolve their disagreements “by themselves” (see Aakhus, 2003; Greco Morasso, 2011; see also Latreille and Saundry, 2014). Moreover, mediation scholars tend to reserve the word “relational” for more “radical” approaches to mediation like (narrative) forms of *transformative mediation*, which aim “to empower the parties, restoring their self-confidence and their responsiveness to others” (Latreille and Saundry, 2014: 192; see also Bush and Folger, 1994; Jameson et al., 2014; Winslade et al., 1998), in contrast to more traditional forms of *facilitative mediation*, which stress “the importance of recognizing the needs and interests of disputants in order to identify areas for agreement that will be sustainable in the future” (Latreille and Saundry, 2014: 192). Our article expands this literature by highlighting the communicative work through which the relations between the disputants, as part of a wider web of relations between the mediator, disputants, and textual actors, gets constituted in the course of mediation, showing that *any* kind of conflict mediation work is a relational interplay of human and (frequently) textual actors’ vector effects.

Second, in line with the previous, this article shows that written texts are more than mere tools or instruments (see also Kameo and Whalen, 2015; Smith, 1990) that institutional representatives, citizens, lawyers, and mediators use “at will” in their communication to accomplish particular things during mediation sessions. They are also more than textual agents merely *acting on behalf of* others (see Brummans, 2007; Cooren, 2004, 2008, 2009). As conjunctive or disjunctive vectors, we have shown, they have the ability to transform the nature of relations together with human actors (the disputants, their legal representatives, etc.) in ways that are advantageous or disadvantageous to one or both of the disputing parties during mediation. Hence, our research reveals how the vector effects of texts are inextricably linked to the vector effects of human beings in mediation work, and how textual and human actors enable and constrain such effects.

In the first case we analyzed, our analysis showed how the nature of the relation between Doug and the institution-via-R1 was constituted through a battle of textual vectors, especially ones that carried or conveyed Dr. D.’s voice differently, thus constituting the disputants’ relation in antagonistic ways. Our vectorial perspective revealed that the vector effects of textual actors such as Dr. D.’s medical expertise report (and thus Dr. D. by proxy) depended for an important part on the vector effects of the human actors through which the texts could speak and act. In this case, we did not obtain data that showed whether Doug actually decided to take his case to court. However, all signs were pointing to an impasse—one that was for an important part created through the aforementioned battle.

In the second case, a new textual actor (a letter to be written by Curt’s doctor) was expected to help resolve the dispute by acting with, for, and through (Cooren, 2018a) the disputing parties in such a way that their key incompatibility became a compatibility. Even though this letter did not exist on paper yet, our analysis showed how increasing its (degree of) existence during the session started to shape the relations between Curt, his lawyer, the institutional representative, and the mediator, and to help lay down a viable path forward. The to-be-written letter began to exist through the human actors and their respective contributions, which allowed them to define its content. Consequently, this letter-in-becoming became *the* vector through which their conflict could be resolved, because it became the common ground on which the participants could act. As in the first case, our vectorial perspective revealed how the letter and human beings exchanged properties. In this second case, however, the exchange enabled them to act together, rather than against each other. While the letter enabled the human actors to get rid of an important obstacle (the outlier report) that had prevented them from moving forward, they enabled this letter to come into existence—and hopefully to exist *more*, depending on whether Curt’s doctor agreed to write it. Unfortunately, we did not obtain data that showed whether this new letter ended up doing what the disputants, lawyer, and mediator hoped it would do in the follow-up session, but things looked quite promising in the session we analyzed.

Thus, our study shows that particular texts are not inherently conjunctive or disjunctive in mediation; their ability to make a difference (agency) as vectors—their vector effects—depends on the relational web or field as it unfolds in the course of communication between different actors. This insight has important implications beyond the context of mediation for scholarship on communicative relationality and, more generally, relational ontologies. As we have shown, conceptualizing actors as vectors and analyzing their effects not only reveal how relational fields of vector effects are communicatively constituted, but also how the different types of actors themselves are formed relationally; how they are *relational beings*, or how their (or any) “*being is relation*,” as Debaise (2004: 16, emphasis in original, our translation) notes, referring to philosophers like Simondon, Spinoza, Nietzsche, Bergson, and Tarde, who came to the same conclusion. Our vectorial perspective therefore provides a new relational view of agency (see Brummans, 2018) that is useful for investigating how the agency of textual and possibly other actors is accomplished in connection with the agency of human actors, and how these interconnections are key to the constitution of relational webs of a multitude of actors.

To conclude, our article suggests that it would be useful for conflict management professionals to broaden their conception of conflict management by trying to find creative ways to mobilize texts, or to prevent them from working against disputants. Regarding the latter, our research indicates that while institutional representatives, lawyers, and mediators often recognize the potential vector effects of texts, as well as the vector effects of their own actions, citizens struggle to perceive it. Many citizens, that is, are not as equipped as institutional representatives or lawyers in mobilizing texts. Mediators can try to intervene to readjust this issue, thus jeopardizing their impartiality. However, other ways could be explored to address this commonly encountered asymmetry between the disputing parties. For example, in pre-mediation sessions, conflict coaches could meet citizens to help them prepare for mediation by, among other things, raising their awareness of texts’ vector effects, as well as their own, thereby bridging this gap in knowledgeability and skill.

As indicated, our methodological decision to focus on conciliation-type mediation limits the transferability (Lincoln and Guba, 1985) of our findings to other types of mediation work and other contexts. Conciliation resembles a court situation in which citizens challenge public institutions’ prior decisions, so written texts “naturally” carry more weight. As vectors, texts nevertheless also play a central role in other types of mediation work, such as divorce or workplace mediation. We noted at the start of this article that the latter is increasingly attracting the attention of researchers and professionals as a valuable alternative form of conflict management in organizations (see Bollen et al., 2016; Latreille and Saundry, 2014). The reason for this popularity, Latreille and Saundry (2014: 190) state, “stems from dissatisfaction with conventional rights-based disputes procedures viewed as cumbersome, inefficient, and adversarial.” Workplace mediation is, in turn, believed to “restore the employment relationship”, offer “significant financial savings compared with often lengthy traditional grievance and disciplinary processes”, “[deliver] sustainable outcomes and high rates of dispute resolution and satisfaction among the parties”, and “trigger the development of improved conflict-handling skills . . . enhance employer–employee relationships . . . and develop organizational culture.” Scholars in fields ranging from organization studies to sociology and communication studies are starting to “make documents a research topic . . . and treat them as a fundamental phenomenon of (and participant in) organizational life” (Kameo and Whalen 2015: 206). So far, however, few studies have investigated how written texts (performance appraisal reports, grievance reports, memos, etc.) help compose the nature of relations between employees/workers, supervisors, line managers, HR practitioners, and so on. The vectorial perspective we have developed here addresses this issue by providing a useful communicative lens for examining the interplay of textual and human actors’ vector effects in forms of organizational conflict management like workplace mediation.
